# Indications Inviting Primary and Secondary Sutures of War Wounds Determined by Bacteriological Investigation

**Published:** 1917-11

**Authors:** 


### *Indications Inviting Primary and Secondary Sutures of War Wounds Determined by Bacteriological Investigation.*

By Georges GROSS, Correspondant National, and H. TISSIER. Trans. and abstracted from the *Bulletins et Mémoires de la Société de Chirurgie de Paris*, July 10, 1917.

The authors note the marked tendency in war surgery towards large incisions and thorough surgical cleansing of wounds, a technique that G. claims to have been among the first to practice and recommend. Ether washing has supplemented this treatment and has proved the best antiseptic method. Excisions now greatly exceed the limits of the altered tissues. The success of this treatment soon led to primitive sutures of war wounds. Loubat, of the authors' ambulance, being the first to put this method into practice for closing wounds of the joints.

Of 44 wounds sutured primarily, 40 resulted in reunions by the first intention. 4 resulted in partial disunions. These results were obtained not only in soft parts but also where the wound was accompanied by fracture.

Determining Considerations :

1. Time elapsed since the reception of the wound (not more than 12 to 18 hours at most).

2. Possibility of making complete and correct excision of damaged tissues and of extracting the projectiles.

3. Clinical judgment of the favorable aspect of lesions.

Although in several cases the authors have been obliged to reopen wounds, they have never had fatal or even serious results.

Sometimes they have effected secondary sutures at the end of 15 or 20 days in the case of apyretic patients in order to hasten healing. It must be admitted that in neither primary nor secondary sutures is it possible to lay down precise indications inviting sutures. Bacteriological tests are the chief guide, but in practicing them one must be influenced by the kind rather than the number of bacteria found in the wounded area.

As to bacteriological tests the authors come to the following conclusions :

1. Each war wound has a special bacterial flora varying often in different wounds on the same patient.

2. Each microbe flourishing on a wound follows its own evolution.

3. Infection is sometimes primary and sometimes secondary. Frequent handling of wounded soldiers increases the chances of multiple infection, and argues for primary and secondary sutures as early as possible.

4. Infection of war wounds often assumes the putrid type because of the anaerobes of putrefaction which flourish only under the following conditions : 1. The existence of a contused tissue or one deprived of circulation : 2. The simultaneous presence of one or more aerobes.

5. The extension of the anaerobic gangrenous process depends upon the aerobe present which seems to prepare the way. Thus only in the case of the common saprophytes, which are only slightly pathogenic, this putrid anaerobic fermentation is localized; with pyogenic staphylococci, it extends slowly; with genuine streptococci, it extends so fast as to be overwhelming.

6. Infection of war wounds may also assume the *purulent* type when the wound has not been infected by anaerobes or when these bacteria have not been able to develop. Here again the individuality of the aerobe gives to the wound a special development. Common saprophytes cause little reaction; staphylococci cause more or less lively reaction: but streptococci give rise to *general* and *local* reaction. In this last case interminable suppuration occurs, remote abcesses, protracted bony lesions, quick temperature changes, and that slow cachexia which undermines the patient.

It is, then, especially necessary in putrid or purulent infection of wounds to know the nature of the aerobe in order to determine the prognosis. The authors lay down the following rules :

1. Practice primitive suture regularly if the removal of the projectile and the excision of devitalized tissue is possible, noting in the meantime the nature of the aerobes contained in the primary discharges. If these are common saprophytes, do not disturb the sutures. If they are staphylococci, watch the wound carefully for slow putrefaction, which is easily removable. If they are genuine streptococci, the stitches should be removed, and thorough excisions, followed by adequate washing, should be made in order to remove all putrid fermentation.

2. When the primitive suture has not been made, effect a secondary one as rapidly as possible, guided only by aerobes. Only when there is an association of anaerobes and streptococci is it necessary to leave war wounds wide open.

A considerable number of cases are given in detail.

